# A Case of Inverted Papilloma Originating from the Middle Ear and Review of the Literature

**DOI:** 10.1155/2019/3041570

**Published:** 2019-07-24

**Authors:** Eray Bayindir, Fatih Mehmet Hanege, Mahmut Tayyar Kalcioglu, Tulay Zenginkinet, Serdal Celik

**Affiliations:** ^1^Istanbul Medeniyet University Faculty of Medicine, Department of Otorhinolaryngology, Istanbul, Turkey; ^2^Istanbul Medeniyet University Faculty of Medicine, Department of Pathology, Istanbul, Turkey

## Abstract

Inverted papilloma (IP) with malignant transformation is rarely seen in the middle ear. Up to now, there have been 16 primary middle ear IPs reported in the English literature. Even though it is very rare in the middle ear, this pathology should be kept in mind for the differential diagnosis of middle ear masses. In this case, we report a 77-year-old female who had recurrent IP with malignant transformation and complication.

## 1. Introduction

Inverted papilloma (IP) is a rare benign tumor that can show recurrence. It constitutes 0.5–4% of nasal tumors [1–3]. It mainly originates from the lateral nasal wall near the middle meatus and often extends to the paranasal sinus [1]. This lesion is locally invasive and has a tendency toward malignant transformation [3, 4]. Inverted papilloma in the middle ear and mastoid cavity might occur extremely rarely as a primary lesion or as an extension of a sinonasal papilloma [3]. IP may have a high degree of dysplasia, and 2–27% of patients may develop invasive squamous cell carcinoma (SCC) [5, 6]. The recurrence and malignancy rates of IP in the middle ear are higher than the rates of sinonasal IP [7]. Radiotherapy or chemotherapy, two of the most common treatment methods, is applied after surgical excision of IP [3]. To date, the number of reported primary IP cases originating from the middle ear and mastoid cavity is 16 in the English literature [4, 7–18]. In this case report, we present a primary middle ear PA and discuss this along with comparisons to reports in the literature.

## 2. Case Presentation

**Figure 1:**
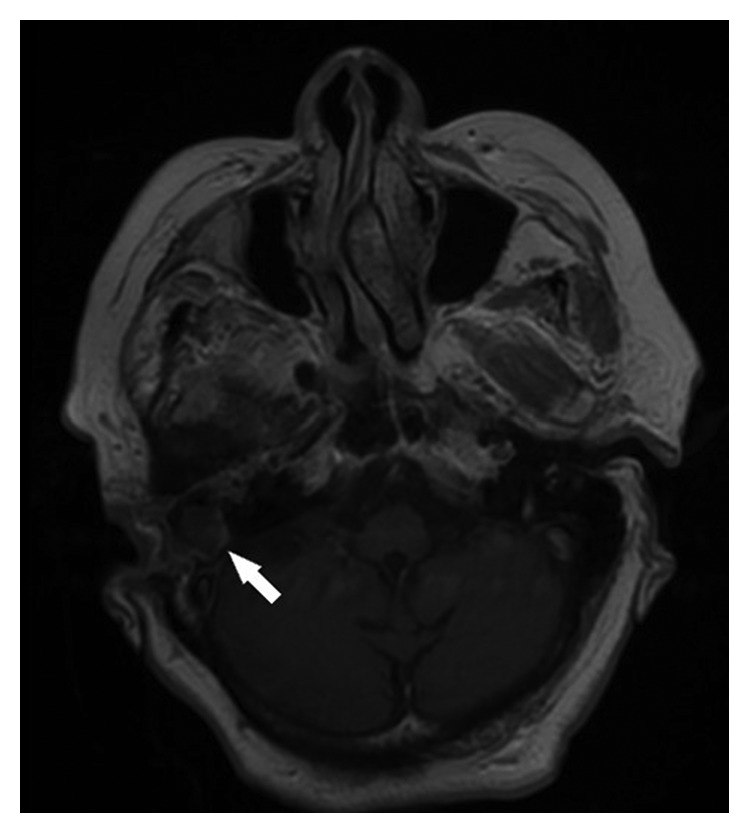
Contrast-enhanced ear MRI axial section. Approximately 2 cm mass showing peripheral contrast enhancement in the right mastoid antrum (white arrow).

**Figure 2:**
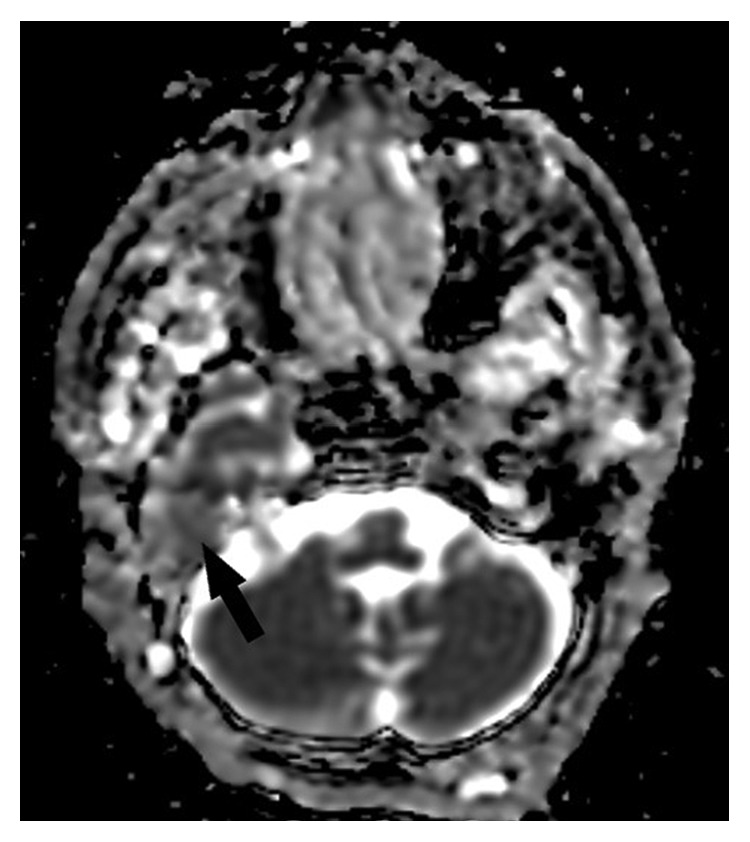
There was no restriction in diffusion-weighted imaging of this lesion (black arrow).

**Figure 3:**
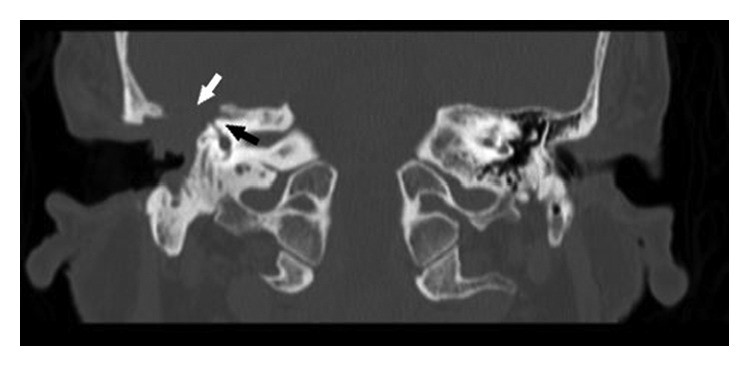
Coronal section of temporal bone CT. A mass obliterates the outer ear pathway and destroys the mastoid cells and mastoid antrum. The mass extends to the middle ear. The mass was caused by ossicular chain and tegmen tympani defects. White arrow: Tegmen tympani defect. Black arrow: defect in superficial semicircular canal.

**Figure 4:**
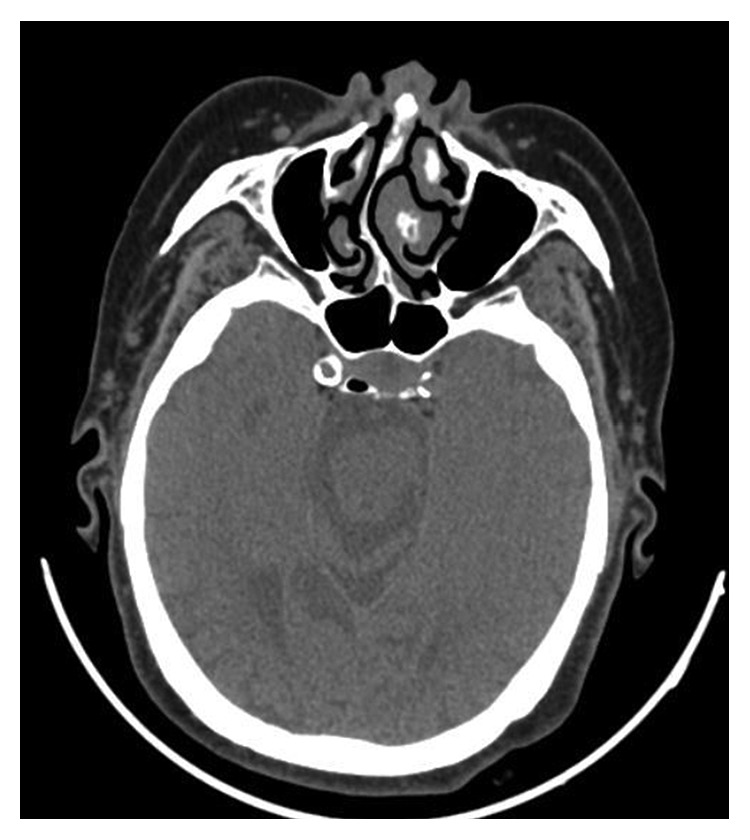
Axial section of temporal bone CT. Bilaterally, the maxillary sinuses have a natural appearance. The nasal cavity has no additional pathology except deviation of the septum.

A 77-year-old female patient who had right tympanomastoidectomy operations twice in another hospital with the diagnosis of chronic otitis media with cholesteatoma was admitted to our clinic with a complaint of discharge in the right ear. The right external auditory canal was filled with a cholesteatomatous mass. On diffusion-weighted temporal bone MRI, soft tissue images showing peripheral contrast enhancement were seen in the right mastoid cells, mastoid antrum, and middle ear. There was no restriction in diffusion-weighted imaging of this lesion, and thus, cholesteatoma was not considered as a diagnosis (Figures 1 and 2). The patient's temporal bone CT images were reported as soft tissue obliterating the right external ear canal, showing extension to the middle ear cavity, causing destruction in the ossicular chain, mastoid antrum, tegmen tympani, and mastoideum (Figure 3). No sinonasal pathology was found in the patient's history. Examination and imaging of the sinonasal region showed no additional pathology (Figure 4). There was advanced sensorineural hearing loss on audiologic evaluation. The patient reported having had hearing loss for many years. In the left ear, both bone conduction and air conduction were 37 dB. Canal wall-down tympanomastoidectomy was performed under general anesthesia. All pathological tissues were cleaned, and because there were no perioperative or postoperative problems, the patient was discharged on postoperative day 2 and asked to return for a routine follow-up visit. The histopathological diagnosis was granulation tissue.

About 6 months after the operation, the patient came back because of recurrence of ear discharge. There was a cholesteatomatous mass observed by otomicroscopic evaluation. Revision surgery was performed, and partial petrosectomy and cavity obliteration of the fatty tissue were done. Histopathologic evaluation of the cleaned tissues was reported as cholesteatoma.

**Figure 5:**
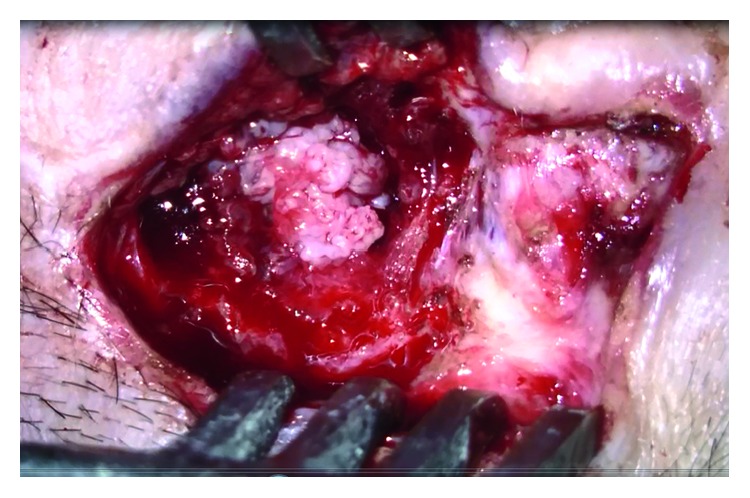
Ulcerovegetative mass filling the entire mastoid area and causing tongue tympanoid erosion (intraoperative view).

**Figure 6:**
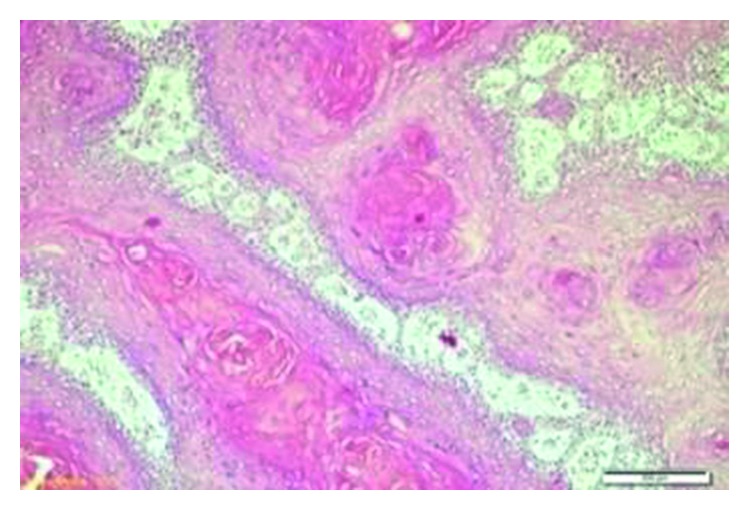
Exophytic papillomatous tumor with a deep endophytic area covered with squamous epithelium, inverted papilloma-high grade squamous dysplasia (H&E, 10x).

Two months after the operation, the patient was admitted to the clinic with a vegetative mass in the postauricular scar area. She also had grade 5 facial paralysis according to the House–Brackmann classification on the right side. The patient was hospitalized again, and revision surgery was performed under general anesthesia. There was an ulcerovegetan mass filling the mastoid area (Figure 5) and eroding bone on the horizontal and vertical facial canal. It was observed that the pathology proceeded to the medial line of the petrous bone. Since the dura was completely exposed, the neurosurgery team was included in the operation. All lesions were cleared, and total petrosectomy was performed. The damaged dura was repaired. The operation was terminated by obliterating the cavity containing the fat tissue. Pathologic evaluation of the specimen was reported as “IP-high grade squamous dysplasia” (Figure 6). The patient received a dose of 16 gy in 8 fractions. Radiotherapy could not be continued because of deterioration of the patient's general medical condition. General health problems worsened, and 4 months after the last operation, the patient passed away due to multiple organ failure.

## 3. Discussion

IP generally originated from the epithelium of paranasal sinuses. [4, 19, 20]. The nasal cavity and paranasal sinuses are covered with a columnar epithelium which is called the Schneiderian epithelium [8, 20]. Thus, a paranasal sinus papilloma is known as a Schneiderian papilloma [6]. IP is the most common subtype of Schneiderian papilloma [7, 19, 20].

**Figure 7:**
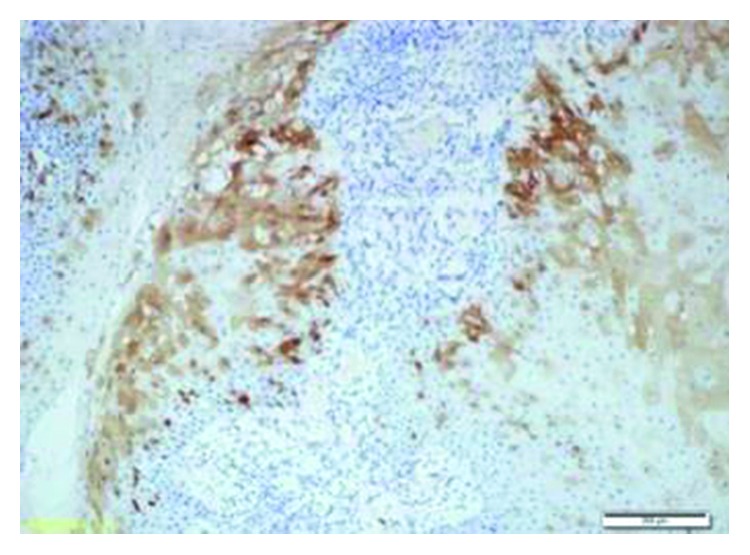
Nuclear and cytoplasmic expressions were observed with p16 (p16, 10x).

**Figure 8:**
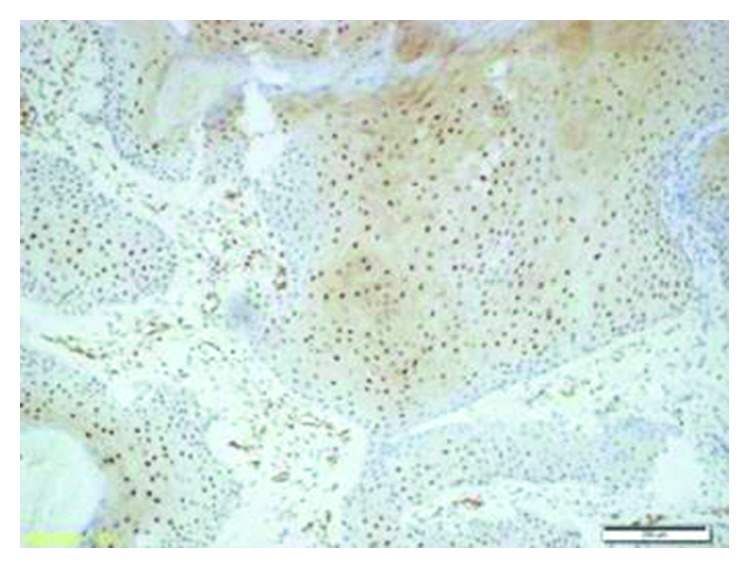
Nuclear and cytoplasmic expressions were observed with p27 (p27, 10x).

The etiology of IP is not clearly understood. Allergic rhinitis, chronic sinusitis, and human papilloma virus (HPV) infection have been discussed as etiologic factors [1, 3]. Blandamura et al. investigated some sex hormones in IPs that had formed in paranasal sinuses and temporal bones and reported progesterone receptor expression in some of these cases [9]. Although there was no progesterone receptor expression, p16 and p27 genes were positive in the current case (Figures 7 and 8).

IP is a locally aggressive tumor, and transformation to squamous cell carcinoma may be seen [21–23]. Vorasubin et al. reported SCC in 3 of 70 cases (8%) with inverted papilloma originating from sinonasal origins. Dysplastic changes also were present in 3 (8%) of the patients [24]. In another study by Barnes, the incidence of malignancy was reported as 11% [25]. The histopathological diagnosis of the current case was IP-high grade squamous dysplasia.

Middle ear or mastoid cavity IP may be in 2 forms. Sinonasal IP may extend into the middle ear, or it may be middle ear-based without any history of sinonasal IP [26]. Primary IPs originating from the mastoid cavity or middle ear are extremely rare [27]. To date, other than our case, the number of cases reported as middle ear- or mastoid-based IP is 16 in the English literature (Table 1). If sinonasal IPs with middle ear involvement are included, this number increases to 39.

The mean age of these 17 primary middle ear- or mastoid-originated cases is 51 years. Malignant transformation occurred in 23.5% of cases, and the recurrence rate was 17.6% in these 17 patients. These rates are higher than the rates which were reported as 7% for malignant transformation of sinonasal IP and 15% for the recurrence rate of sinonasal IP [19]. Both recurrence and malignant transformation were seen in the current case.

Although the pathogenesis of IP in the middle ear and the mastoid cavity is not clearly understood, two hypotheses have been discussed. One of them is direct extension of sinonasal pathology, and the other is abnormally displaced embryonic Schneiderian membrane into the middle ear [12]. In the current case, since no pathological appearance on evaluation of the sinonasal zone could be detected, it was thought that the disease had developed as a result of abnormal embolization of embryonic Schneiderian membrane into the middle ear.

Surgery is the preferred method of treatment for middle ear and mastoid IP [2, 3, 19]. It is difficult to define a standard surgical strategy based on the limited data available in the literature. Tympanomastoidectomy, radical temporal bone resection, or petrosectomy may be preferred depending on the location of the disease [2]. Total petrosectomy was performed in our last surgery, where the case was diagnosed as IP. Radiotherapy could be added if the tumor is not fully resected or if there is more than one recurrence, even without malignancy [2]. Since our patient had both recurrence and malignant transformation, we planned complementary treatment with radiotherapy. However, the general health status of the patient did not allow for completion of radiotherapy, and she died from multiorgan failure.

In conclusion, even though it is very rare, IP can be seen in the middle ear. This pathology with local recurrence and malignant transformation potential should be considered in the differential diagnosis of recurrent middle ear diseases.

## Figures and Tables

**Table 1:** Inverted papilloma cases and features of primary middle ear and mastoid origin published in the literature.

No.	Literature reference	Age	Sex	Location	MT		Treatment		Recurrence	
					Yes	No	Surg	RT	Yes	No
1	Roberts et al. [11]	19	F	ME		+	+		+	
2	Wenig [12]	31	F	ME		+	+			+
3	Wenig [12]	56	F	MC, ME		+	+			+
4	Wenig [12]	19	F	ME		+	+			+
5	Wenig [12]	57	F	ME		+	+			+
6	Chhetri et al. [13]	26	F	EAC, ME		+	+		+	
7	de Filippis et al. [15]	58	M	MC, ME		+	+			+
8	Blandamura et al. [9]	54	F	ME		+	+		NA	NA
9	Cahali et al. [10]	72	M	ME, EAC		+	+			+
10	Ali et al. [14]	42	F	MC, ME, EAC	NA	NA	+			+
11	Zhou et al. [16]	52	M	MC, ME, EAC	+		+			+
12	van der Putten et al. [7]	74	F	MC, EAC		+	+		NA	NA
13	Coca-Pelaz et al. [4]	74	M	ME		+	+		NA	NA
14	Schaefer et al. [18]	46	M	ME, MC		+	+			+
15	Nath and Das [17]	60	M	MC	+		+	+		+
16	Mummadi et al. [8]	60	M	MC, ME	+		+	+	NA	NA
17	Current case	77	F	MC, ME, EAC	+		+	+	+	

M: male; F: female; MC: mastoid cavity; ME: middle ear; EAC: external auditory canal; RT: radiotherapy; MT: malignant transformation; Surg: surgery; RT: radiotherapy; N/A: not applicable.

## References

[1] Shen J., Baik F., Mafee M. F., Peterson M., Nguyen Q. T. (2011). Inverting papilloma of the temporal bone. *Otology & Neurotology*.

[2] Barbosa J. L., Pinheiro S. D., de Freitas M. R., Nunes A. A. A., Leite E. B. (2012). Sinonasal inverted papilloma involving the middle ear and the mastoid. *Brazilian Journal of Otorhinolaryngology*.

[3] Acevedo-Henao C.-M., Talagas M., Marianowski R., Pradier O. (2010). Recurrent inverted papilloma with intracranial and temporal fossa involvement: a case report and review of the literature. *Cancer/Radiothérapie*.

[4] Coca-Pelaz A., Gómez-Martínez J., Vivanco-Allende B., Hermsen M., Llorente J. L. (2016). Primary inverted papilloma of the middle ear with intracranial invasion. *Head & Neck*.

[5] Lawson W., Kaufman M. R., Biller H. F. (2003). Treatment outcomes in the management of inverted papilloma: an analysis of 160 cases. *The Laryngoscope*.

[6] Krouse J. H. (2001). Endoscopic treatment of inverted papilloma: safety and efficacy. *American Journal of Otolaryngology*.

[7] van der Putten L., Bloemena E., Merkus P., Hensen E. F. (2013). Schneiderian papilloma of the temporal bone. *Case Reports*.

[8] Mummadi S. M., Darr A., Hakim N., Din S., Bhimrao S. K. (2018). A rare case of Schneiderian papilloma of the middle ear presenting with pulsatile tinnitus. *Annals of the Royal College of Surgeons of England*.

[9] Blandamura S., Marioni G., de Filippis C., Giacomelli L., Segato P., Staffieri A. (2003). Temporal bone and sinonasal inverted papilloma. *Archives of Otolaryngology—Head & Neck Surgery*.

[10] Cahali S., Silva F. B. D., Machado M. C., Silva D. A. D., Reforeme O. M. R., Cahali M. B. (2005). Papiloma escamoso de orelha média: relato de um caso e revisão da literatura. *Revista Brasileira de Otorrinolaringologia*.

[11] Roberts W. H., Dinges D. L., Hanly M. G. (1993). Inverted papilloma of the middle ear. *Annals of Otology, Rhinology & Laryngology*.

[12] Wenig B. M. (1996). Schneiderian-type mucosal papillomas of the middle ear and mastoid. *Annals of Otology, Rhinology & Laryngology*.

[13] Chhetri D. K., Gajjar N. A., Bhuta S., Andrews J. C. (2001). Pathology forum. quiz case 2. schneiderian-type papilloma of the middle ear. *Archives of Otolaryngology—Head & Neck Surgery*.

[14] Ali R. B., Amin M., Hone S. (2011). Tinnitus as an unusual presentation of Schneiderian papillomatosis. *Irish Journal of Medical Science*.

[15] de Filippis C., Marioni G., Tregnaghi A., Marino F., Gaio E., Staffieri A. (2002). Primary inverted papilloma of the middle ear and mastoid. *Otology & Neurotology*.

[16] Zhou H., Chen Z., Li H., Xing G. (2011). Primary temporal inverted papilloma with premalignant change. *Journal of Laryngology & Otology*.

[17] Nath J., Das B. (2016). Primary inverted papilloma of middle ear and mastoid: a rare case report. *Journal of Clinical and Diagnostic Research*.

[18] Schaefer N., Chong J., Griffin A., Little A., Gochee P., Dixon N. (2015). Schneiderian-type papilloma of the middle ear: a review of the literature. *International Surgery*.

[19] Rubin F., Badoual C., Moya-Plana A., Malinvaud D., Laccourreye O., Bonfils P. (2012). Inverted papilloma of the middle ear. *European Annals of Otorhinolaryngology, Head and Neck Diseases*.

[20] Eggers G., Mühling J., Hassfeld S. (2007). Inverted papilloma of paranasal sinuses. *Journal of Cranio-Maxillofacial Surgery*.

[21] O’Connell B. P., Rivas A., Wanna G. B., Haynes D. S. (2010). Inverting papilloma of the middle ear: a case report. *The Laryngoscope*.

[22] Seshul M. J., Eby T. L., Crowe D. R., Peters G. E. (1995). Nasal inverted papilloma with involvement of middle ear and mastoid. *Archives of Otolaryngology—Head and Neck Surgery*.

[23] Durucu C., Baglam T., Karatas E., Mumbuc S., Kanlikama M. (2009). Surgical treatment of inverted papilloma. *Journal of Craniofacial Surgery*.

[24] Vorasubin N., Vira D., Suh J. D., Bhuta S., Wang M. B. (2013). Schneiderian papillomas: comparative review of exophytic, oncocytic, and inverted types. *American Journal of Rhinology & Allergy*.

[25] Barnes L., Barnes L. (2009). Diseases of the nasal cavity, paranasal sinuses, and nasopharynx. *Surgical Pathology of the Head and Neck*.

[26] Carlson M. L., Sweeney A. D., Modest M. C., Van Gompel J. J., Haynes D. S., Neff B. A. (2015). Inverting papilloma of the temporal bone: report of four new cases and systematic review of the literature. *The Laryngoscope*.

[27] Kaddour H. S., Woodhead C. J. (1992). Transitional papilloma of the middle ear. *Journal of Laryngology & Otology*.

